# The Obesity Paradox in Hepatocellular Carcinoma: Insights from Continuous and Interaction-Based Analyses of Body Mass Index After Hepatic Resection

**DOI:** 10.3390/cancers18071143

**Published:** 2026-04-02

**Authors:** Boram Lee, Ho-Seong Han, Yoo-Seok Yoon, Jai Young Cho, Hae Won Lee, Yeshong Park, Hyelim Joo, Seung Yeon Lim

**Affiliations:** 1 Department of Surgery, Seoul National University Bundang Hospital, Seoul National University College of Medicine, Seoul 03080, Republic of Korea; boramsnubhgs@gmail.com (B.L.); yoonys@snubh.org (Y.-S.Y.); jychogs@gmail.com (J.Y.C.); lansh@hanmail.net (H.W.L.); yeshong.park@gmail.com (Y.P.); kaylin-j@snubh.org (H.J.); symangolim@gmail.com (S.Y.L.); 2 Department of Surgery, Seongnam Citizen Medical Center, Seongnam 13411, Republic of Korea

**Keywords:** hepatocellular carcinoma, hepatic resection, body mass index, obesity paradox, overall survival, recurrence-free survival, prognostic factors

## Abstract

**Background/Objectives**: The prognostic significance of body mass index (BMI) in hepatocellular carcinoma (HCC) remains controversial, and whether the obesity paradox may reflect tumor biology or host-related factors is unclear. This study evaluated the association between BMI and survival after curative hepatic resection using continuous and interaction-based analyses. **Methods:** We retrospectively analyzed 1349 patients who underwent curative hepatic resection for HCC between 2004 and 2021. BMI was assessed both categorically (low (<18.5 kg/m^2^), normal (18.5–24.9 kg/m^2^), and high (≥25 kg/m^2^)) and as a continuous variable. Overall survival (OS) and recurrence-free survival (RFS) were analyzed using Kaplan–Meier methods and multivariable Cox proportional hazards models. Interaction terms were incorporated to examine whether the prognostic effect of BMI varied across clinically relevant subgroups defined by tumor differentiation, tumor size, tumor number, and alpha-fetoprotein level. **Results:** OS differed significantly across BMI categories (log-rank *p* < 0.001), whereas differences in RFS were modest. At 3 years, estimated OS rates were 88%, 82%, and 62% in the high, normal, and low BMI groups, respectively. In multivariable analysis, higher BMI as a continuous variable was independently associated with improved OS (hazard ratio per 1-unit increase, 0.87; 95% confidence interval, 0.79–0.95; *p* = 0.005), but not with RFS. Subgroup analyses demonstrated a consistent protective association between BMI and OS without significant interactions across tumor-related factors. **Conclusions:** Higher BMI is independently associated with improved overall survival after hepatic resection for HCC, irrespective of tumor biology. These findings support a host-related explanation for the obesity paradox in surgically treated HCC.

## Simple Summary

Body weight and nutritional status are known to influence outcomes in many cancers, but their role in hepatocellular carcinoma (HCC) remains unclear. Some studies suggest that patients with higher body mass index (BMI) may live longer, a phenomenon known as the obesity paradox, but it is uncertain whether this reflects differences in tumor behavior or patient-related factors. In this study, we examined the relationship between BMI and survival after hepatic resection. By analyzing BMI as a continuous measure, we evaluated whether its association with survival was influenced by tumor characteristics. We found that patients with higher BMI had better survival after surgery, regardless of tumor size, grade, or biological aggressiveness, while body mass index was not strongly related to cancer recurrence. These findings suggest that BMI may reflect host-related factors affecting long-term survival rather than tumor behavior itself and may help improve postoperative risk assessment in patients undergoing liver cancer surgery.

## 1. Introduction

Hepatocellular carcinoma (HCC) is the most common primary liver cancer and ranks as the third leading cause of cancer-related mortality worldwide [1,2]. Despite advances in surveillance strategies, surgical techniques, and perioperative management, long-term outcomes after curative hepatic resection remain highly variable, largely due to the heterogeneous nature of tumor biology and underlying liver function [3,4,5]. Even after curative resection, recurrence rates of HCC remain substantial, underscoring the importance of identifying reliable prognostic factors that extend beyond conventional tumor-related variables [5].

Numerous tumor- and liver-related factors—including tumor size, multiplicity, vascular invasion, satellite nodules, serum alpha-fetoprotein (AFP) level, and the presence of cirrhosis—have been consistently associated with oncologic outcomes following hepatic resection for HCC [6,7,8,9,10]. These factors have been incorporated into widely accepted staging systems and treatment algorithms [11,12]. However, increasing attention has been directed toward host-related factors, particularly nutritional and metabolic status, as potentially modifiable determinants of long-term survival [13,14,15].

Body mass index (BMI), a widely used surrogate marker for nutritional status, has been linked to prognosis in various malignancies [16]. While low BMI is generally associated with poor survival, the prognostic implications of higher BMI remain controversial [17]. Paradoxically, several studies have suggested improved survival among overweight or obese patients, a phenomenon termed the “obesity paradox” [18]. However, evidence supporting this paradox in hepatocellular carcinoma (HCC) is limited and inconsistent, and most prior studies have relied on categorical BMI classifications without adequately examining whether BMI-related survival effects are consistent across clinically relevant subgroups defined by tumor burden or biological aggressiveness.

In this context, we aimed to evaluate the prognostic impact of BMI on long-term outcomes after curative hepatic resection for HCC. We analyzed the association between BMI and OS and RFS using BMI as a continuous variable, assessed its independent prognostic value in multivariable models, and examined the consistency of BMI-related effects across clinically meaningful subgroups using interaction-based analyses.

## 2. Materials and Methods

### 2.1. Study Design and Patient Selection

This retrospective cohort study was conducted at Seoul National University Bundang Hospital. Data were retrieved from a prospectively maintained database of patients who underwent hepatic resection for HCC between 2004 and 2021. Among 1784 initially identified patients, those with R1 resection margins, preoperative spontaneous tumor rupture, or incomplete clinical data were excluded. Finally, 1349 patients with complete clinicopathological and preoperative staging data were included for analysis.

### 2.2. Preoperative Assessment

All patients underwent standard preoperative evaluation, including contrast-enhanced triphasic computed tomography (CT) and/or magnetic resonance imaging (MRI) to assess tumor characteristics such as size, number, vascular invasion, and extrahepatic spread. Liver functional reserve was evaluated using conventional laboratory parameters, including serum bilirubin, albumin, international normalized ratio (INR), and platelet count, and summarized using the Child–Pugh classification. Decisions regarding the extent of liver resection were based on tumor location, estimated remnant liver volume, and the presence of portal hypertension.

### 2.3. Surgical Procedure

The extent of liver resection was classified according to the Brisbane 2000 terminology [19]. Anatomical resection was performed using standard inflow and outflow control techniques to remove the tumor-bearing segments. Non-anatomical limited resections were selectively performed for peripherally located tumors, particularly in patients with cirrhotic liver disease, at the surgeon’s discretion.

### 2.4. Data Collection and Variables

Demographic, clinical, surgical, pathological, and laboratory data were collected. Liver function was assessed using the Child–Pugh classification. Tumor characteristics were evaluated according to the Edmondson-Steiner grading and the 8th edition AJCC TNM staging system [20]. Body mass index (BMI) was calculated as weight in kilograms divided by height in meters squared (kg/m^2^). For descriptive analyses, BMI was categorized according to World Health Organization criteria as low (<18.5 kg/m^2^), normal (18.5–24.9 kg/m^2^), and high (≥25 kg/m^2^) [21]. For survival analyses, BMI was additionally modeled as a continuous variable to evaluate its dose-dependent prognostic effect. For survival analyses, other key variables included age, sex, serum albumin, INR, platelet count, tumor size, tumor number, presence of satellite nodules, microvascular invasion, macrovascular invasion, growth pattern, Edmondson grade, and serum alpha-fetoprotein (AFP) level. AFP was dichotomized at 200 ng/mL based on established prognostic thresholds.

### 2.5. Definition of Outcomes

Overall survival (OS) was defined as the time from surgery to death from any cause or last follow-up. Recurrence-free survival (RFS) was defined as the time from surgery to the first documented tumor recurrence. Patients who died without evidence of recurrence were censored at the time of death. The final follow-up date was January 2025, ensuring a minimum potential follow-up of three years for all patients.

### 2.6. Statistical Analysis

Categorical variables are presented as numbers and percentages and were compared using the chi-square test or Fisher’s exact test, as appropriate. Continuous variables are expressed as median with interquartile range and were compared using the Mann–Whitney U test or Kruskal–Wallis test. OS and RFS were estimated using the Kaplan–Meier method and compared using the log-rank test. The prognostic impact of BMI on OS and RFS was evaluated using Cox proportional hazards regression analyses. The proportional hazards assumption was evaluated by visual inspection of log-minus-log survival plots, and no major violations were observed. Univariable analyses were first performed, followed by multivariable Cox models incorporating clinically relevant covariates. BMI was primarily analyzed as a continuous variable in multivariable models to assess its independent and dose-dependent association with survival outcomes. Results are reported as hazard ratios (HRs) with 95% confidence intervals (CIs). To examine whether the prognostic effect of BMI varied across clinically relevant subgroups, interaction analyses were conducted by including interaction terms between BMI (continuous) and predefined subgroup variables in multivariable Cox models. Subgroups were defined according to tumor differentiation (Edmondson grade 1–2 vs. ≥3), tumor size (≤5 cm vs. >5 cm), tumor number (single vs. multiple), and AFP level (<200 vs. ≥200 ng/mL). Statistical significance of interactions was assessed using the corresponding *p* values for interaction. All statistical analyses were performed using IBM SPSS Statistics for Windows, v25.0 (IBM Corp., Armonk, NY, USA), and a two-sided *p* value < 0.05 was considered statistically significant. Multicollinearity among variables was assessed using variance inflation factors.

## 3. Results

### 3.1. Baseline Characteristics According to BMI Category

Baseline clinicopathological characteristics according to BMI category are summarized in Table 1. Among the 1349 patients included in the analysis, 35 (2.6%) had low BMI, 752 (55.8%) had normal BMI, and 562 (41.6%) had high BMI. Male patients were more frequently observed in the high BMI group compared with the normal and low BMI groups (50.9% vs. 35.6% and 14.3%, respectively; *p* < 0.001). The prevalence of hypertension was also significantly higher in patients with high BMI (32.7%) than in those with normal (24.1%) or low BMI (22.9%) (*p* = 0.004). Serum albumin levels differed modestly across BMI categories, with slightly lower values observed in the low BMI group (*p* = 0.043), whereas age, diabetes mellitus, prior abdominal surgery, previous transarterial chemoembolization or radiofrequency ablation, Child–Pugh class, viral etiology, MELD score, and platelet count were comparable among the three groups. Importantly, tumor-related characteristics—including tumor size, presence of cirrhosis, and the extent of surgical resection—did not significantly differ according to BMI category. The proportions of patients undergoing anatomical resection, laparoscopic surgery, and major hepatectomy were also similar across BMI groups.

### 3.2. Survival Outcomes According to BMI Category

**Figure 1:**
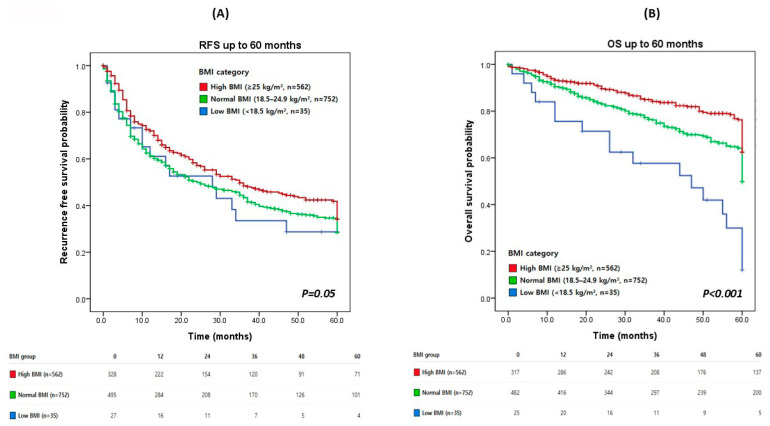
Kaplan–Meier overall survival curves according to BMI group. Kaplan–Meier curves illustrating (**A**) recurrence-free survival (RFS) and (**B**) overall survival (OS) according to body mass index (BMI) category in patients undergoing curative hepatic resection for hepatocellular carcinoma. Patients were stratified into low BMI (<18.5 kg/m^2^), normal BMI (18.5–24.9 kg/m^2^), and high BMI (≥25 kg/m^2^) groups. Differences between groups were assessed using the log-rank test. Numbers at risk are shown below each plot.

Kaplan–Meier analyses demonstrated significant differences in RFS and OS according to BMI category (Figure 1). Kaplan–Meier analyses showed a modest association between BMI category and RFS (Figure 1A). Although patients with high BMI tended to have more favorable RFS compared with those with normal or low BMI, the differences among BMI groups were limited and reached only borderline statistical significance (log-rank *p* = 0.05). At 3 years after surgery, the estimated RFS rates were approximately 55% in the high BMI group, 48% in the normal BMI group, and 40% in the low BMI group, with less pronounced and less consistent separation of survival curves over time. In contrast, BMI category was strongly associated with OS (Figure 1B). Patients with high BMI demonstrated the most favorable OS, followed by those with normal BMI, whereas patients with low BMI experienced the poorest survival outcomes (log-rank *p* < 0.001). At 3 years after surgery, the estimated OS rates were approximately 88%, 82%, and 62% in the high, normal, and low BMI groups, respectively, demonstrating a clear stepwise gradient across BMI categories. This separation of survival curves persisted throughout the follow-up period, with consistently higher OS observed in patients with high BMI.

### 3.3. Multivariable Cox Regression Analysis for Survival Outcomes

**Figure 2:**
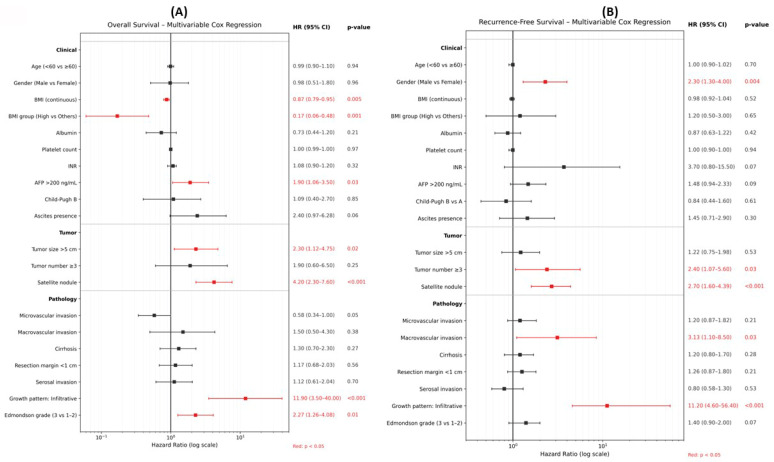
Multivariable Cox proportional hazards analysis for survival outcomes. Forest plots showing multivariable Cox proportional hazards regression analyses for (**A**) overall survival (OS) and (**B**) recurrence-free survival (RFS) after curative hepatic resection for hepatocellular carcinoma. Hazard ratios (HRs) with 95% confidence intervals (CIs) are presented on a logarithmic scale. BMI was analyzed both as a continuous variable and as a categorical variable (high BMI vs. others). Models were adjusted for clinically relevant demographic, liver function-related, and tumor-related covariates. Variables with *p* < 0.05 are highlighted in red.

Multivariable Cox proportional hazards analysis identified BMI as an independent prognostic factor for OS (Figure 2A). When modeled as a continuous variable, higher BMI was significantly associated with improved OS (hazard ratio [HR] per 1-unit increase, 0.87; 95% confidence interval [CI], 0.79–0.95; *p* = 0.005). Consistently, patients in the high BMI group demonstrated a significantly lower risk of mortality compared with those in the other BMI categories (HR, 0.17; 95% CI, 0.06–0.48; *p* = 0.001). In contrast, age and sex were not independently associated with OS. Among tumor-related factors, tumor size >5 cm (HR, 2.30; 95% CI, 1.12–4.75; *p* = 0.023), presence of satellite nodules (HR, 4.20; 95% CI, 2.30–7.60; *p* < 0.001), infiltrative growth pattern (HR, 11.90; 95% CI, 3.50–40.00; *p* < 0.001), and higher Edmondson grade (3 vs. 1–2; HR, 2.27; 95% CI, 1.26–4.08; *p* = 0.01) were independently associated with worse OS. Liver function-related variables, including Child–Pugh class and cirrhosis, were not significant predictors in the multivariable model.

In contrast to OS, BMI was not independently associated with RFS in multivariable analysis, whether analyzed as a continuous variable (HR, 0.98; 95% CI, 0.92–1.04; *p* = 0.52) or as a categorical variable (high BMI vs others; HR, 1.20; 95% CI, 0.50–3.00; *p* = 0.65) (Figure 2B). Instead, recurrence risk was primarily driven by tumor-related factors, including tumor number ≥3 (HR, 2.40; 95% CI, 1.07–5.60; *p* = 0.033), presence of satellite nodules (HR, 2.70; 95% CI, 1.60–4.39; *p* < 0.001), macrovascular invasion (HR, 3.13; 95% CI, 1.10–8.50; *p* = 0.025), and infiltrative growth pattern (HR, 11.20; 95% CI, 4.60–56.40; *p* < 0.001).

### 3.4. Subgroup Analysis of BMI on Overall Survival

**Figure 3:**
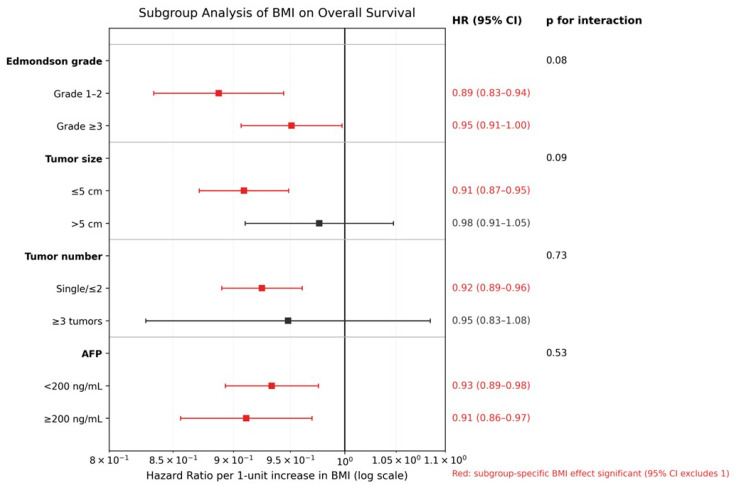
Subgroup analysis of BMI on overall survival. Forest plot illustrating subgroup analyses of the association between body mass index (BMI) and overall survival (OS). Hazard ratios (HRs) with 95% confidence intervals (CIs) represent the effect per 1-unit increase in BMI and are displayed on a logarithmic scale. Subgroup analyses were performed according to Edmondson grade (1–2 vs. ≥3), tumor size (≤5 cm vs. >5 cm), tumor number (single/≤2 vs. ≥3), and serum alpha-fetoprotein (AFP) level (<200 vs. ≥200 ng/mL). Interaction terms between BMI and each subgroup variable were included in multivariable Cox models, and corresponding *p* values for interaction are shown. Red symbols indicate subgroup-specific BMI effects that reached statistical significance (95% CI excluding 1).

Subgroup analyses were performed to evaluate whether the prognostic impact of BMI on OS differed according to key tumor-related and pathological factors (Figure 3). When BMI was analyzed as a continuous variable, higher BMI was consistently associated with a reduced risk of mortality across all examined subgroups. Specifically, the protective effect of higher BMI on OS was observed in patients with both well to moderately differentiated tumors (Edmondson grade 1–2; HR per 1-unit increase, 0.89; 95% CI, 0.83–0.94) and poorly differentiated tumors (grade ≥3; HR, 0.95; 95% CI, 0.91–1.00), with no statistically significant interaction between BMI and tumor differentiation (*p* for interaction = 0.08). Similarly, BMI demonstrated a favorable association with OS in patients with tumors ≤5 cm (HR, 0.91; 95% CI, 0.87–0.95) as well as those with tumors >5 cm (HR, 0.98; 95% CI, 0.91–1.05), without evidence of effect modification by tumor size (*p* for interaction = 0.09).

The association between BMI and OS also remained consistent irrespective of tumor number, with comparable effects observed in patients with single or limited tumors and those with multiple tumors (*p* for interaction = 0.73). Likewise, higher BMI was associated with improved OS in both patients with low AFP levels (<200 ng/mL; HR, 0.93; 95% CI, 0.89–0.98) and those with elevated AFP levels (≥200 ng/mL; HR, 0.91; 95% CI, 0.86–0.97), with no significant interaction detected (*p* for interaction = 0.53). Overall, no significant interactions were identified between BMI and any of the predefined subgroup variables, indicating that the favorable association between higher BMI and overall survival was broadly consistent across clinically relevant subgroups.

## 4. Discussion

In this large single-center cohort of patients undergoing curative hepatic resection for hepatocellular carcinoma, we demonstrated that body mass index (BMI) was independently associated with overall survival (OS) but not with recurrence-free survival (RFS). Higher BMI was consistently associated with improved OS across multiple analytical approaches, including categorical comparisons, continuous modeling, and multivariable Cox regression. Importantly, this favorable association persisted across clinically relevant subgroups without significant interaction, indicating that the prognostic impact of BMI on OS was broadly consistent regardless of tumor burden or biological aggressiveness.

A key finding of the present study is the dissociation between OS and RFS with respect to BMI. While patients with higher BMI exhibited significantly improved OS, BMI was not independently associated with RFS in multivariable analyses. This distinction suggests that the survival advantage associated with higher BMI is unlikely to be driven by reduced tumor recurrence. Rather, BMI may reflect host-related factors influencing overall survival; however, given the lack of association with recurrence-free survival, this interpretation should be made with caution, and the underlying mechanisms remain unclear [6,9,10,11,12]. Alternative explanations, including reverse causation due to cancer-related weight loss, occult cachexia or systemic inflammation, and potential lead-time bias, should also be considered. Surgical approaches, including the extent of resection and use of minimally invasive techniques, were comparable across BMI groups, suggesting that differences in surgical management are unlikely to explain the observed survival outcomes. These findings underscore the importance of differentiating OS from RFS when evaluating host-related prognostic factors in HCC, as reliance on recurrence-based endpoints alone may obscure clinically meaningful survival effects.

Baseline characteristics further support this interpretation. Tumor-related variables and surgical extent were well balanced across BMI categories, minimizing the likelihood that differences in tumor burden or treatment intensity accounted for the observed survival advantage in patients with higher BMI. In contrast, host-related factors differed among BMI groups. Patients with low BMI exhibited lower serum albumin levels, suggesting reduced nutritional reserve, whereas patients with high BMI more frequently had metabolic comorbidities such as hypertension. Notably, despite a higher prevalence of metabolic risk factors, patients with higher BMI demonstrated superior OS, reinforcing the concept that nutritional or physiological reserve may outweigh the negative effects of metabolic comorbidity in the postoperative course of HCC patients [22,23,24,25].

The consistent protective association between higher BMI and OS across subgroup analyses further strengthens this conclusion. The favorable effect of BMI was observed irrespective of tumor differentiation, tumor size, tumor number, or AFP level, and no significant interactions were identified. These findings argue against the notion that the prognostic relevance of BMI is confined to selected low-risk populations and instead suggest a generalized host-related survival benefit [18]. The absence of effect modification by tumor-related factors also supports the biological plausibility that BMI influences survival through mechanisms distinct from intrinsic tumor aggressiveness.

Our findings align with the growing body of literature describing the so-called “obesity paradox”, in which overweight or obese patients experience improved survival outcomes despite an increased burden of comorbidities [18]. In the context of HCC, previous studies have reported conflicting results regarding the prognostic role of BMI, often limited by heterogeneous patient populations, reliance on categorical BMI classifications, and inadequate adjustment for confounders. By modeling BMI as a continuous variable and incorporating interaction-based subgroup analyses, the present study provides a more nuanced and robust assessment of BMI-related survival effects following hepatic resection.

Several mechanisms may underlie the observed association between higher BMI and improved OS. Higher BMI may serve as a surrogate marker of preserved nutritional status, greater metabolic reserve, or enhanced tolerance to surgical stress and subsequent treatments. Patients with higher BMI may better withstand postoperative complications, disease progression, or salvage therapies after recurrence. Conversely, low BMI may reflect sarcopenia, frailty, or systemic inflammation, all of which have been associated with adverse outcomes in HCC. Although BMI does not directly capture body composition, its consistent association with OS in this study suggests that it retains prognostic relevance in routine clinical practice [26,27,28,29].

The present study has several limitations. Its retrospective design introduces the potential for residual confounding despite comprehensive multivariable adjustment. In addition, the long study period may introduce bias related to temporal changes in surgical techniques, perioperative management, and treatment strategies, as well as residual confounding from unmeasured variables such as performance status, detailed liver function indices, comorbidity burden, and post-recurrence treatment strategies. These factors may have influenced survival outcomes. In addition, overall survival may be influenced by non-cancer-related mortality, particularly in patients with chronic liver disease. The lack of cause-specific mortality data limits the ability to fully assess the oncologic impact of BMI; therefore, the observed association should be interpreted with caution. BMI was used as a surrogate for nutritional status and body composition, and measures such as sarcopenia or visceral adiposity were not available. Moreover, BMI is an imperfect surrogate that does not distinguish between muscle mass, fat distribution, or fluid retention, particularly in patients with chronic liver disease. CT-based body composition parameters, including skeletal muscle mass, were not consistently available in this cohort, precluding further analysis of sarcopenia. Even if BMI reflects underlying conditions such as sarcopenia, frailty, or poor nutritional status, it may still serve as a practical clinical marker in routine settings. However, BMI remains a simple and universally available parameter in routine clinical practice, and its independent association with overall survival suggests that it may still capture clinically meaningful aspects of patient condition. In addition, BMI was assessed at a single preoperative time point, and changes in body weight over time were not captured, which may limit the ability to evaluate the dynamic impact of nutritional status. Additionally, the number of patients in the low BMI group was relatively small, which may limit the stability of categorical comparisons. However, BMI was primarily analyzed as a continuous variable to mitigate potential bias related to unequal group sizes and to better capture its dose-dependent association with outcomes. The relatively large effect size observed in categorical comparisons should therefore be interpreted with caution, and the continuous BMI analysis likely provides a more robust estimate of the association. In addition, interaction analyses may have been underpowered due to the relatively small number of patients in certain subgroups, and the absence of statistically significant interactions should therefore be interpreted with caution. In addition, interaction analyses may have been underpowered due to the relatively small number of patients in certain subgroups. Furthermore, these analyses were exploratory in nature and not pre-specified, and no formal power calculation for interaction testing was performed. Therefore, the absence of statistically significant interactions, as well as borderline findings, may reflect limited statistical power and the possibility of type II error. Finally, this was a single-center study, which may limit generalizability.

## 5. Conclusions

In conclusion, higher BMI was independently and consistently associated with improved overall survival after curative hepatic resection for hepatocellular carcinoma, while no independent association with recurrence-free survival was observed. These findings suggest that BMI may reflect host-related factors that influence long-term survival rather than tumor recurrence itself. Consideration of BMI and nutritional status may therefore provide valuable prognostic information and help refine risk stratification in patients undergoing surgical treatment for HCC.

## Figures and Tables

**Table 1:** Patient population.

	Total (*n* = 1349)	Low BMI (<18.5 kg/m^2^) N = 35	Normal BMI (18.5–24.9 kg/m^2^) N = 752	High BMI (≥25 kg/m^2^) N = 562	*p* Value
Male (*n*, %)	559 (41.4%)	5 (14.3%)	268 (35.6%)	286 (50.9%)	<0.001
Age (mean ± SD)	60.7 ± 11.0	63.0 ± 12.9	60.7 ± 11.2	60.6 ± 10.7	0.894
* BMI (mean ± SD (range))	24.5 ± 3.4 (17.7–31.3)	17.1 ± 2.5 (12.1–18.4)	22.6 ± 1.6 (18.5–24.9)	27.5 ± 2.4 (25.0–32.3)	-
HTN (*n*, %)	373 (27.6%)	8 (22.9%)	181 (24.1%)	184 (32.7%)	0.004
DM (*n*, %)	422 (31.3%)	11 (31.4%)	218 (29.0%)	193 (34.3%)	0.138
Previous abdomen op hx (*n*, %)	362 (26.8%)	14 (40.0%)	203 (27.0%)	145 (25.8%)	0.143
Previous TACE (*n*, %	157 (11.6%)	4 (11.4%)	83 (11.0%)	70 (12.5%)	0.796
Previous RFA (*n*, %)	51 (3.8%)	3 (8.6%)	26 (3.5%)	22 (3.9%)	0.264
Child–Pugh score (*n*, %)					0.569
A	1301 (96.4%)	32 (91.4%)	727 (96.7%)	542 (96.4%)	
B	48 (3.6%)	3 (8.6%)	25 (3.3%)	20 (3.6%)	
Virology (*n*, %)					
HBV	927 (60.8%)	25 (73.5%)	474 (70.2%)	347 (67.1%)	0.437
HCV	84 (5.5%)	2 (5.9%)	46 (6.8%)	30 (5.8%)	0.772
MELD (mean ± SD)	7.8 ± 2.5	6.0 ± 1.3	8.0 ± 1.6	7.6 ± 2.5	0.153
Albumin (mean ± SD)	4.2 ± 0.4	4.1 ± 0.4	4.2 ± 0.4	4.2 ± 0.5	0.043
Plt (mean ± SD)	171 ± 27.5	149.3 ± 58.5	176.4 ± 70.6	175.3 ± 63.9	0.153
AFP (median [IQR])	54.9 (0–2482)	72.0 (0–7120)	14.2 (0–7580)	85.2 (0–770)	0.549
Tumor size (mean ± SD)	3.1 ± 2.0	2.7 ± 2.0	3.3 ± 1.8	3.2 ± 1.2	0.645
Cirrhosis (*n*, %)	811 (60.1%)	23 (65.7%)	455 (60.5%)	333 (59.3%)	0.445
Anatomical resection (*n*, %)	709 (52.6%)	21 (60.0%)	406 (54.0%)	282 (50.2%)	0.318
Laparoscopy (*n*, %)	952 (70.6%)	22 (62.9%)	511 (68.0%)	419 (74.6%)	0.516
Major resection (*n*, %)	271 (20.10%)	7 (20.0%)	168 (22.3%)	96 (17.1%)	0.554

HTN, hypertension; DM, diabetes mellitus; TACE, transarterial chemoembolization; RFA, radiofrequency ablation; HBV, hepatitis B virus; HCV, hepatitis C virus; MELD, model for end-stage liver disease; Plt, platelet count; AFP, alpha-fetoprotein; SD, standard deviation. Percentages are calculated within each BMI group. * BMI was used to define the groups and therefore not compared statistically.

## Data Availability

The data that support the findings of this study are available from the corresponding author upon reasonable request. Due to privacy restrictions, the data are not publicly available.

## Abbreviations

HCC	Hepatocellular carcinoma
BMI	Body mass index
OS	Overall survival
RFS	Recurrence-free survival
AFP	Alpha-fetoprotein
LLR	Laparoscopic liver resection
RFA	Radiofrequency ablation
TACE	Transarterial chemoembolization
AJCC	American Joint Committee on Cancer
BCLC	Barcelona Clinic Liver Cancer
